# Supplementary material for: Effect of Second-Hand Smoke Exposure on Establishing Urinary Cotinine-Based Optimal Cut-Off Values for Smoking Status Classification in Korean Adults
Source: Int J Environ Res Public Health. 2022 Jun 29;19(13):7971. doi: 10.3390/ijerph19137971 (PMC9265992; doi:10.3390/ijerph19137971)
Supplement: Supplementary file 1 [file ijerph-19-07971-s001.zip › ijerph-1783241-supplementary.pdf]

Supplementary Table S1. Dataset recruitment

Dataset	Survey project	Year	Survey Institution
2008 KNHANES	The Fourth Korea National Health and Nutrition Examination Survey (KNHANES IV-2)	2008	Korea Centers for Disease Control and Prevention
2011 KNHANES	The Fifth Korea National Health and Nutrition Examination Survey (KNHANES V-2)	2011	Korea Centers for Disease Control and Prevention
2014 KNHANES	The Sixth Korea National Health and Nutrition Examination Survey (KNHANES VI-2)	2014	Korea Centers for Disease Control and Prevention
2018 KNHANES	The Seventh Korea National Health and Nutrition Examination Survey (KNHANES VII-3)	2018	Korea Centers for Disease Control and Prevention

Access link https://knhanes.kdca.go.kr/knhanes/sub03/sub03_02_05.do

Supplementary Table S2. Raw variant codes related with self-report for smoking status classification in all datasets

Variant code	Description	Questionnaire	Subvariant	Subvariant status
BS1_1	Life smoking status	How many cigarettes have you smoked in your lifetime?	1	Less than 5 packs (100 cigarettes)
			2	Equal to or more than 5 packs (100 cigarettes)
			3	Never smoked
BS3_1	current smoking status	Do you currently smoke?	1	Yes, I smoke at least one cigarette a day
			2	Yes, I smoke, but not every day
			3	No, but I smoked in the past
			8	Not included (BS1_1=3 or <19 years)
			9	Unknown/No response for self-report
sm_presnt	current smoking rate		0	Non-smoker, former smoker
			1	Current smoker (BS1_1=2 and [BS3_1=1 or BS3_1=2)

Supplementary Table S3. Data processing related with self-report for smoking status classification in all datasets

Smoking status	Description	Variant code	Processing
Current smoker	a subject who reported a history of 100 cigarettes smoking in his or her lifetime reported currently smoking cigarettes	sm_presnt	sm_presnt=1
Non-smoker	a subject who did not meet the current smoker definition among responded subjects for self-report for smoking status	sm_presnt	sm_presnt=0
Daily smoker	a subject who reported "Yes, I smoke at least one cigarette a day", among current smokers	BS3_1, sm_presnt	BS3_1=1 and sm_presnt=1
Non-daily smoker	a subject who reported "Yes, I smoke, but not every day", among current smokers.	BS3_1, sm_presnt	BS3_1=2 and sm_presnt=1

Supplementary Table S4. Raw variant codes related with self-report for SHS exposure status classification in each dataset

Dataset	Variant code	Description	Questionnaire	Subvariant	Subvariant status
2008	BS8_2	SHS exposure at indoor workplace	How much did you inhale other's cigarette smoke indoors at work, in the past 7 days?	1	zero hour
				2	Less than one hour
				3	Equal to or more than one hour
				9	Unknown/No response for self-report
	BS9_2	SHS exposure at home	How much did you inhale other's cigarette smoke indoors at home, in the past 7 days?	1	zero hour
				2	Less than one hour
				3	Equal to or more than one hour
				8	Not included (<19 years, no one in the family regularly smokes indoors at home)
				9	Unknown/No response for self-report
2011	BS8_2	SHS exposure at indoor workplace	How much did you inhale other's cigarette smoke indoors at work, in the past 7 days?	1	zero hour
				2	Less than one hour
				3	Equal to or more than one hour

				9	Unknown/No response for self-report
	BS9_2	SHS exposure at home	How much did you inhale other's cigarette smoke indoors at home, in the past 7 days?	1	zero hour
				2	Less than one hour
				3	Equal to or more than one hour
				8	Not included (<19 years, no one in the family regularly smokes indoors at home)
				9	Unknown/No response for self-report
2014	BS8_2	SHS exposure at indoor workplace	Have you been inhaling other's cigarette smoke indoors at work, in the past 7 days?	1	Yes
				2	No
				8	Not working
				9	Unknown/No response for self-report
	BS9_2	SHS exposure at home	Have you been inhaling other's cigarette smoke indoors at home, in the past 7 days?	1	Yes
				2	No
				8	No one in the family regularly smokes indoors at home, except for himself or herself.
				9	Unknown/No response for self-report

2018	BS8_2	SHS exposure at indoor workplace	Have you been inhaling other's cigarette smoke indoors at work, in the past 7 days?	1	Yes
				2	No
				8	Not working
				9	Unknown/No response for self-report
	BS9_2	SHS exposure at home	Have you been inhaling other's cigarette smoke indoors at home, in the past 7 days?	1	Yes
				2	No
				3	No one in the family regularly smokes indoors at home, except for himself or herself.
				8	Not included (<6 years)
				9	Unknown/No response for self-report

Supplementary Table S5. Data processing related with self-report for SHS exposure status classification in each dataset

Dataset	SHS exposure status	Description	Variant code	Processing
2008	SHS exposure	a subject who reported a history of SHS exposure at home or at indoor workplace among non-smokers	BS8_2, BS9_2, sm_presnt	(BS8_2=2 and sm_presnt=0) or (BS8_2=3 and sm_presnt=0) or (BS9_2=2 and sm_presnt=0) or (BS9_2=3 and sm_presnt=0)
	Unclear response	a subject who is not included in SHS exposure and who reported unclear history of SHS exposure, such as no response, unknown response, and non-defined response, at home or at indoor workplace among non-smokers	BS8_2, BS9_2, sm_presnt	Step 1 selection (BS8_2=9 and sm_presnt=0) or (BS9_2=9 and sm_presnt=0) Step 2 remove from selection (BS8_2=2 and sm_presnt=0) or (BS8_2=3 and sm_presnt=0) or (BS9_2=2 and sm_presnt=0) or (BS9_2=3 and sm_presnt=0)
	No SHS exposure	a subject who definitively reported no history of SHS exposure at home and at indoor workplace among non-smokers	BS8_2, BS9_2, sm_presnt	[(BS8_2=1 and sm_presnt=0) or (BS8_2=8 and sm_presnt=0)] and [(BS9_2=1 and sm_presnt=0) or (BS9_2=8 and sm_presnt=0)]

2011	SHS exposure	a subject who reported a history of SHS exposure at home or at indoor workplace among non-smokers	BS8_2, BS9_2, sm_presnt	(BS8_2=2 and sm_presnt=0) or<br>(BS8_2=3 and sm_presnt=0) or<br>(BS9_2=2 and sm_presnt=0) or<br>(BS9_2=3 and sm_presnt=0)
	Unclear response	a subject who is not included in SHS exposure and who reported unclear history of SHS exposure, such as no response, unknown response, and non-defined response, at home or at indoor workplace among non-smokers	BS8_2, BS9_2, sm_presnt	Step 1 selection<br>(BS8_2=9 and sm_presnt=0) or<br>(BS9_2=9 and sm_presnt=0)<br>Step 2 remove from selection<br>(BS8_2=2 and sm_presnt=0) or<br>(BS8_2=3 and sm_presnt=0) or<br>(BS9_2=2 and sm_presnt=0) or<br>(BS9_2=3 and sm_presnt=0)
	No SHS exposure	a subject who definitively reported no history of SHS exposure at home and at indoor workplace among non-smokers	BS8_2, BS9_2, sm_presnt	[(BS8_2=1 and sm_presnt=0) or<br>(BS8_2=8 and sm_presnt=0)] and<br>[(BS9_2=1 and sm_presnt=0) or<br>(BS9_2=8 and sm_presnt=0)]
2014	SHS exposure	a subject who reported a history of SHS exposure at home or at indoor workplace among non-smokers	BS8_2, BS9_2, sm_presnt	(BS8_2=1 and sm_presnt=0) or<br>(BS9_2=1 and sm_presnt=0)

	Unclear response	a subject who is not included in SHS exposure and who reported unclear history of SHS exposure, such as no response, unknown response, and non-defined response, at home or at indoor workplace among non-smokers	BS8_2, BS9_2, sm_presnt	Step 1 selection<br>(BS8_2=9 and sm_presnt=0) or<br>(BS9_2=9 and sm_presnt=0)<br>Step 2 remove from selection<br>(BS8_2=1 and sm_presnt=0) or<br>(BS9_2=1 and sm_presnt=0)
	No SHS exposure	a subject who definitively reported no history of SHS exposure at home and at indoor workplace among non-smokers	BS8_2, BS9_2, sm_presnt	[(BS8_2=2 and sm_presnt=0) or<br>(BS8_2=8 and sm_presnt=0)] and<br>[(BS9_2=2 and sm_presnt=0) or<br>(BS9_2=8 and sm_presnt=0)]
2018	SHS exposure	a subject who reported a history of SHS exposure at home or at indoor workplace among non-smokers	BS8_2, BS9_2, sm_presnt	(BS8_2=1 and sm_presnt=0) or<br>(BS9_2=1 and sm_presnt=0)
	Unclear response	a subject who is not included in SHS exposure and who reported unclear history of SHS exposure, such as no response, unknown response, and non-defined response, at home or at indoor workplace among non-smokers	BS8_2, BS9_2, sm_presnt	Step 1 selection<br>(BS8_2=9 and sm_presnt=0) or<br>(BS9_2=8 and sm_presnt=0) or<br>(BS9_2=9 and sm_presnt=0)<br>Step 2 remove from selection

			(BS8_2=1 and sm_presnt=0) or (BS9_2=1 and sm_presnt=0)
No SHS exposure	a subject who definitively reported no history of SHS exposure at home and at indoor workplace among non-smokers	BS8_2, BS9_2, sm_presnt	[(BS8_2=2 and sm_presnt=0) or (BS8_2=8 and sm_presnt=0)] and [(BS9_2=2 and sm_presnt=0) or (BS9_2=3 and sm_presnt=0)]

Supplementary Table S6. Data processing related with self-report for current e-cigarette user classification in all datasets

Smoking status	Description	Variant code	Processing
Current e-cigarette user	a subject who reported a history of e-cigarette smoking within 30 days	BS12_2	BS12_2=1
Non- e-cigarette user	a subject who did not meet the current e-cigarette user definition among responded subjects for self-report for smoking status	BS12_2	BS12_2=2, or<br>BS12_2=8, or<br>BS12_2=9
Current smoker with usage of e-cigarette	a subject who reported "Yes, I smoked e-cigarette within 30 days", among current smokers	BS12_2, sm_presnt	BS3_1=1 and sm_presnt=1
Non-smoker with usage of e-cigarette	a subject who reported "Yes, I smoked e-cigarette within 30 days", among non-smokers.	BS12_2, sm_presnt	BS3_1=1 and sm_presnt=0

Supplementary Table S7. The measurement procedures and parameters of surveys.

Parameters	KNHANES (2008-2018)			
	2008	2011	2014	2018
Measurement procedure of urinary cotinine	GC-MS/MS	GC-MS/MS	GC-MS/MS	HPLC-MS/MS
LoD (μg/L)	0.25	0.25	0.27	0.27
Participants with equal to or less than LoD of urinary cotinine (n, %)	1129 (20.0%)	114 (6.1%)	202 (4.1%)	1216 (21.1%)
Participants with equal to or less than 0.30 μg/L of urinary cotinine (n, %)	1129 (20.0%)	114 (6.1%)	202 (4.1%)	1216 (21.1%)
LoQ (μg/L)	0.75	0.75	0.82	0.31
Participants with equal to or less than LoQ of urinary cotinine (n, %)	1274 (22.5%)	207 (11.2%)	1373 (27.8%)	1252 (21.7%)
Participants with equal to or less than 0.75 μg/L of urinary cotinine (n, %)	1274 (22.5%)	207 (11.2%)	1222 (24.7%)	3363 (58.2%)

LoD=the limit of detection, LoQ=the limit of quantitation.

Figure Legend

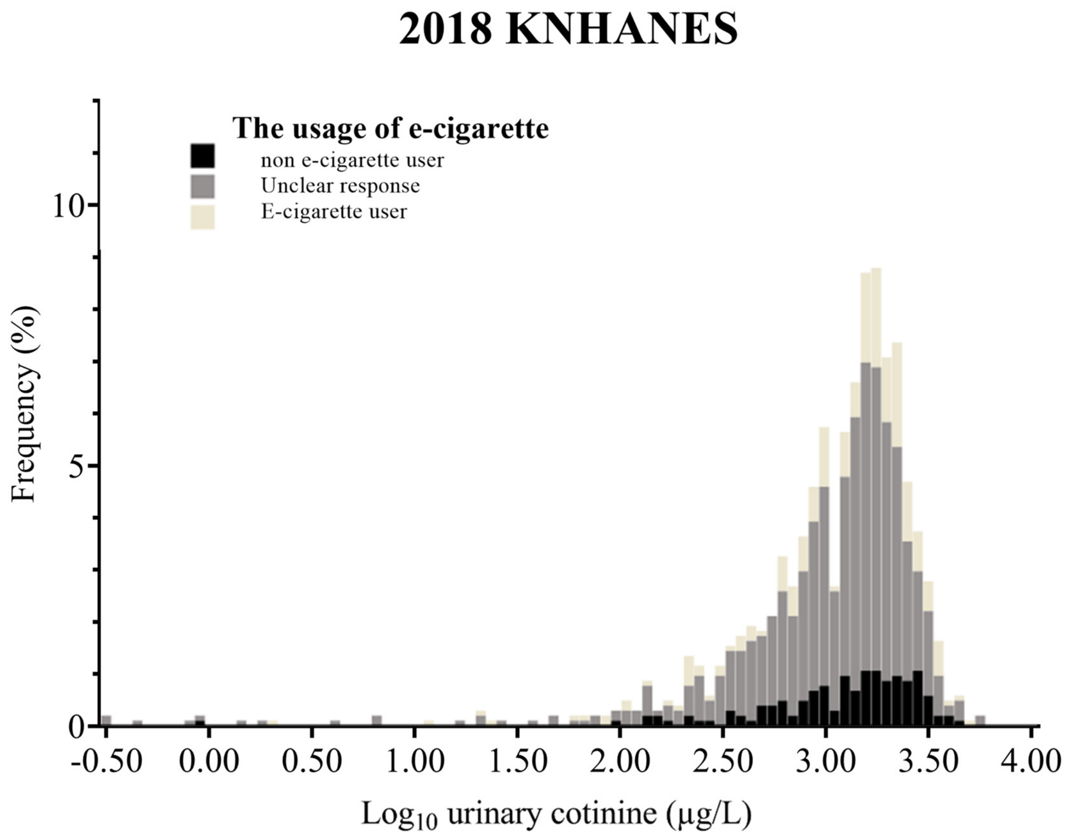

Supplementary Figure S1. Distributions of urinary cotinine concentration according to the usage of e-cigarette among current smokers in 2018 KNHANES

The questionnaire for e-cigarette is only included in 2014 and 2018 KNHANES survey, among four KNHANES datasets.

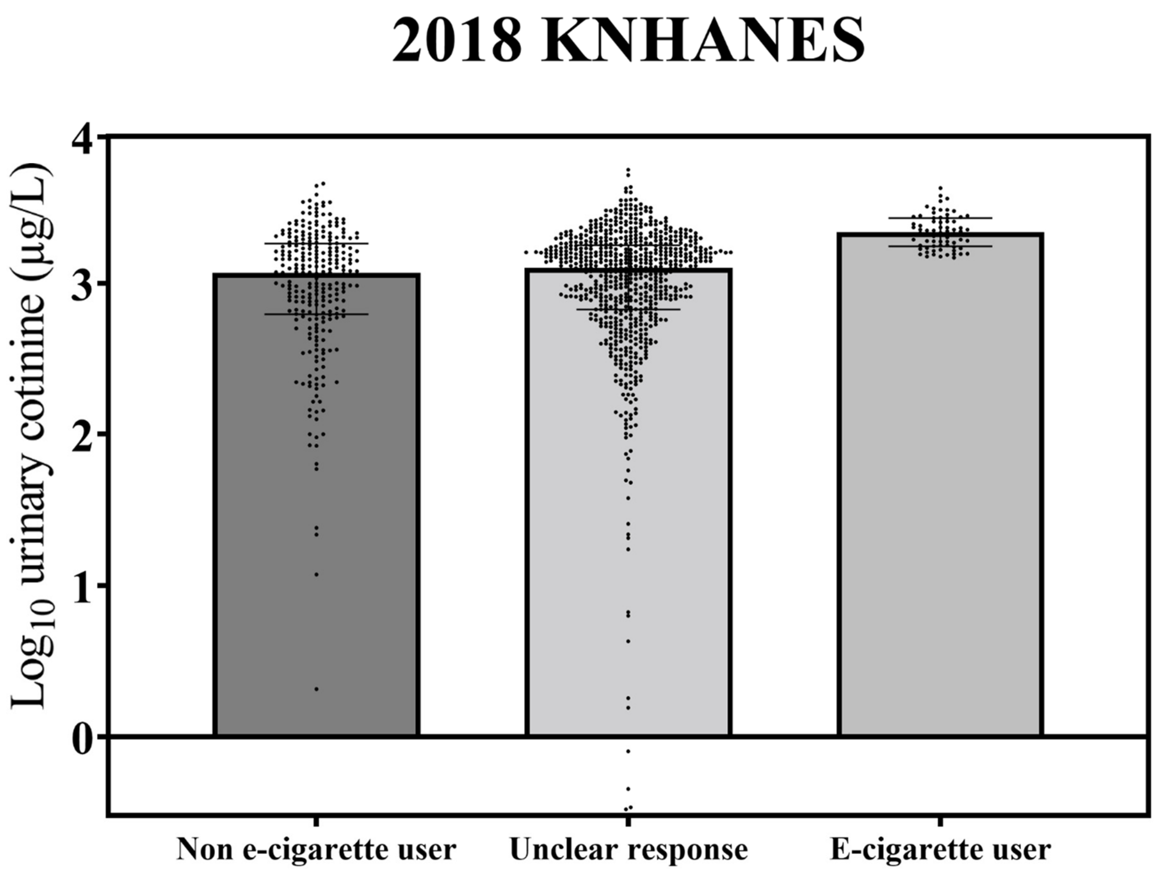

Supplementary Figure S2. The comparison of urinary cotinine concentration according to the usage of e-cigarette among current smokers in 2018 KNHANES

The questionnaire for e-cigarette is only included in 2014 and 2018 KNHANES survey, among four KNHANES datasets.
